# Supplementary material for: Unveiling the Bioactive Potential of the Invasive Jellyfish Phyllorhiza punctata Through Integrative Transcriptomic and Proteomic Analyses
Source: Biomolecules. 2025 Aug 4;15(8):1121. doi: 10.3390/biom15081121 (PMC12383608; doi:10.3390/biom15081121)
Supplement: Supplementary file 1 [file biomolecules-15-01121-s001.zip › Suplementary Material/FileS3/PEPFOLD4/XP_065065372_5520/clusters.html]

 PEP-FOLD clusters


Download archive of all models  here   

|  |  |  |
| --- | --- | --- |
| # | file | sOPEP |
| # cluster 1 - Size: 6 |  |  |
|  | XP\_065065372\_5520-model1 | -7.49935 |
|  | XP\_065065372\_5520-model1.1 | -7.49935 |
|  | XP\_065065372\_5520-model1.2 | -7.49935 |
|  | XP\_065065372\_5520-model1.3 | -7.49935 |
|  | XP\_065065372\_5520-model1.4 | -7.49523 |
|  | XP\_065065372\_5520-model1.5 | -7.49523 |
| # cluster 2 - Size: 83 |  |  |
|  | XP\_065065372\_5520-model2 | -4.06931 |
|  | XP\_065065372\_5520-model2.1 | -4.02376 |
|  | XP\_065065372\_5520-model2.2 | -4.02309 |
|  | XP\_065065372\_5520-model2.3 | -4.02056 |
|  | XP\_065065372\_5520-model2.4 | -4.02056 |
|  | XP\_065065372\_5520-model2.5 | -4.02056 |
|  | XP\_065065372\_5520-model2.6 | -4.01701 |
|  | XP\_065065372\_5520-model2.7 | -4.01595 |
|  | XP\_065065372\_5520-model2.8 | -4.01595 |
|  | XP\_065065372\_5520-model2.9 | -4.01595 |
|  | XP\_065065372\_5520-model2.10 | -4.01595 |
|  | XP\_065065372\_5520-model2.11 | -4.01595 |
|  | XP\_065065372\_5520-model2.12 | -4.00429 |
|  | XP\_065065372\_5520-model2.13 | -4.00429 |
|  | XP\_065065372\_5520-model2.14 | -4.00225 |
|  | XP\_065065372\_5520-model2.15 | -4.00225 |
|  | XP\_065065372\_5520-model2.16 | -3.99292 |
|  | XP\_065065372\_5520-model2.17 | -3.99292 |
|  | XP\_065065372\_5520-model2.18 | -3.99292 |
|  | XP\_065065372\_5520-model2.19 | -3.98013 |
|  | XP\_065065372\_5520-model2.20 | -3.98013 |
|  | XP\_065065372\_5520-model2.21 | -3.98013 |
|  | XP\_065065372\_5520-model2.22 | -3.98013 |
|  | XP\_065065372\_5520-model2.23 | -3.98013 |
|  | XP\_065065372\_5520-model2.24 | -3.98013 |
|  | XP\_065065372\_5520-model2.25 | -3.98013 |
|  | XP\_065065372\_5520-model2.26 | -3.98013 |
|  | XP\_065065372\_5520-model2.27 | -3.98013 |
|  | XP\_065065372\_5520-model2.28 | -3.98013 |
|  | XP\_065065372\_5520-model2.29 | -3.98013 |
|  | XP\_065065372\_5520-model2.30 | -3.98013 |
|  | XP\_065065372\_5520-model2.31 | -3.98013 |
|  | XP\_065065372\_5520-model2.32 | -3.98013 |
|  | XP\_065065372\_5520-model2.33 | -3.98013 |
|  | XP\_065065372\_5520-model2.34 | -3.98013 |
|  | XP\_065065372\_5520-model2.35 | -3.98013 |
|  | XP\_065065372\_5520-model2.36 | -3.98013 |
|  | XP\_065065372\_5520-model2.37 | -3.98013 |
|  | XP\_065065372\_5520-model2.38 | -3.98013 |
|  | XP\_065065372\_5520-model2.39 | -3.98013 |
|  | XP\_065065372\_5520-model2.40 | -3.97779 |
|  | XP\_065065372\_5520-model2.41 | -3.97779 |
|  | XP\_065065372\_5520-model2.42 | -3.97779 |
|  | XP\_065065372\_5520-model2.43 | -3.97779 |
|  | XP\_065065372\_5520-model2.44 | -3.97779 |
|  | XP\_065065372\_5520-model2.45 | -3.97779 |
|  | XP\_065065372\_5520-model2.46 | -3.97779 |
|  | XP\_065065372\_5520-model2.47 | -3.97779 |
|  | XP\_065065372\_5520-model2.48 | -3.97779 |
|  | XP\_065065372\_5520-model2.49 | -3.97779 |
|  | XP\_065065372\_5520-model2.50 | -3.97779 |
|  | XP\_065065372\_5520-model2.51 | -3.97346 |
|  | XP\_065065372\_5520-model2.52 | -3.97346 |
|  | XP\_065065372\_5520-model2.53 | -3.9673 |
|  | XP\_065065372\_5520-model2.54 | -3.9673 |
|  | XP\_065065372\_5520-model2.55 | -3.9673 |
|  | XP\_065065372\_5520-model2.56 | -3.9673 |
|  | XP\_065065372\_5520-model2.57 | -3.9673 |
|  | XP\_065065372\_5520-model2.58 | -3.9673 |
|  | XP\_065065372\_5520-model2.59 | -3.9673 |
|  | XP\_065065372\_5520-model2.60 | -3.9673 |
|  | XP\_065065372\_5520-model2.61 | -3.9673 |
|  | XP\_065065372\_5520-model2.62 | -3.95574 |
|  | XP\_065065372\_5520-model2.63 | -3.95574 |
|  | XP\_065065372\_5520-model2.64 | -3.95574 |
|  | XP\_065065372\_5520-model2.65 | -3.94444 |
|  | XP\_065065372\_5520-model2.66 | -3.94444 |
|  | XP\_065065372\_5520-model2.67 | -3.94444 |
|  | XP\_065065372\_5520-model2.68 | -3.94444 |
|  | XP\_065065372\_5520-model2.69 | -3.94444 |
|  | XP\_065065372\_5520-model2.70 | -3.94444 |
|  | XP\_065065372\_5520-model2.71 | -3.94444 |
|  | XP\_065065372\_5520-model2.72 | -3.94444 |
|  | XP\_065065372\_5520-model2.73 | -3.94195 |
|  | XP\_065065372\_5520-model2.74 | -3.93214 |
|  | XP\_065065372\_5520-model2.75 | -3.92948 |
|  | XP\_065065372\_5520-model2.76 | -3.92948 |
|  | XP\_065065372\_5520-model2.77 | -3.9128 |
|  | XP\_065065372\_5520-model2.78 | -3.89905 |
|  | XP\_065065372\_5520-model2.79 | -3.87516 |
|  | XP\_065065372\_5520-model2.80 | -3.87388 |
|  | XP\_065065372\_5520-model2.81 | -3.87388 |
|  | XP\_065065372\_5520-model2.82 | -3.79358 |
| # cluster 3 - Size: 11 |  |  |
|  | XP\_065065372\_5520-model3 | -3.97786 |
|  | XP\_065065372\_5520-model3.1 | -3.97786 |
|  | XP\_065065372\_5520-model3.2 | -3.97786 |
|  | XP\_065065372\_5520-model3.3 | -3.9654 |
|  | XP\_065065372\_5520-model3.4 | -3.96481 |
|  | XP\_065065372\_5520-model3.5 | -3.96481 |
|  | XP\_065065372\_5520-model3.6 | -3.95473 |
|  | XP\_065065372\_5520-model3.7 | -3.9101 |
|  | XP\_065065372\_5520-model3.8 | -3.9101 |
|  | XP\_065065372\_5520-model3.9 | -3.90614 |
|  | XP\_065065372\_5520-model3.10 | -3.89749 |
|  |  |  |
